# Integrated Microbiome–Metabolome Analysis Reveals Intestine–Liver Metabolic Associations in the Moustache Toad

**DOI:** 10.3390/ani15131973

**Published:** 2025-07-04

**Authors:** Shui-Sheng Yu, Jing-Wen Xiang, Lin Zhang, Xiao-Hua Guo, Yu Wang, Guo-Hua Ding, Hua-Li Hu

**Affiliations:** 1 Laboratory of Amphibian Diversity Investigation, College of Ecology, Lishui University, Lishui 323000, China; schhc@sina.com (S.-S.Y.); jingwenx0530@163.com (J.-W.X.); 2 Administration Center of Zhejiang Jiulongshan National Nature Reserve, Suichang, Lishui 323312, China; yuwang202506@163.com; 3 Hubei Shizhen Laboratory, School of Basic Medical Sciences, Hubei University of Chinese Medicine, Wuhan 430065, China; lzhangss@msn.com; 4 Ecological Forestry Development Center, Suichang, Lishui 323300, China; scguoxiaohua@foxmail.com

**Keywords:** amphibian, intestinal microbiota, gut–liver axis, *Leptobrachium liui*, microbiome–metabolome

## Abstract

The intestinal microbiota regulates host metabolic homeostasis through production of bioactive microbial metabolites. These microorganisms facilitate digestion, enhance immune function, maintain osmoregulation, and support physiological balance via these bioactive compounds, thereby enhancing environmental adaptation. Our study investigated intestinal microbiota–liver metabolic interactions in *Leptobrachium liui* using 16S rRNA gene sequencing and non-targeted liquid chromatography–tandem mass spectrometry metabolomics. Key findings include (1) comparable alpha diversity but distinct microbial community structures between the small intestine (SI) and large intestine (LI), with the SI dominated by Enterobacteriaceae (72.14%) and the LI by Chitinophagaceae (55.16%); (2) segment-specific microbe–metabolite correlations, with predominantly positive correlations in the SI and complex patterns in the LI involving fatty acids, amino acids, and energy metabolites; and (3) significant correlations between specific bacterial families (Aeromonadaceae, Enterobacteriaceae, Chitinophagaceae) and hepatic metabolites related to fatty acid metabolism, amino acid synthesis, and energy pathways, indicating potential gut–liver axis associations. These findings provide insights into amphibian intestinal microbiota–hepatic metabolite associations and may inform future studies of host–microbe interactions.

## Simple Summary

Intestinal microbiota-derived metabolites play crucial roles in modulating host physiological equilibrium through complex microbe–host interactions. This study investigated intestinal microbiota diversity and microbial–metabolite correlations between the small and large intestine of *Leptobrachium liui*, an endemic Chinese amphibian. The results revealed that the small and large intestine harbor distinct microbial community structures despite similar diversity levels. Network analysis demonstrated segment-specific correlations between intestinal microbiota and hepatic metabolites, particularly involving fatty acid, amino acid, and energy metabolism pathways, indicating potential gut–liver axis interactions that may contribute to amphibian physiology.

## 1. Introduction

Maintenance of organismal homeostasis is intrinsically linked to the stability of intestinal microbial communities, with the intestinal microbiota playing a pivotal role in preserving physiological equilibrium [1]. A diverse and balanced intestinal microbiota orchestrates systemic physiological functions through its metabolic output, enabling the host to maintain normal physiological balance despite external perturbations [2]. Through microbial fermentation of non-digestible dietary components, intestinal microbes produce various bioactive metabolites, including short-chain fatty acids (SCFAs), bile acids, and tryptophan derivatives, which collectively influence not only energy metabolism, but also immune modulation, neurological function, and organ health maintenance [3]. More specifically, SCFAs enhance intestinal barrier function by promoting tight junction protein expression in epithelial cells, while simultaneously regulating immune responses and attenuating inflammation [4]. Bile acid metabolites modulate lipid metabolism and hepatic function through nuclear receptor signaling pathways [5]. Tryptophan metabolites mediate intestine–brain axis communication, influencing cognitive function and emotional regulation [6,7]. Perturbation of this microbial ecosystem (dysbiosis) leads to metabolic imbalance, subsequently compromising homeostasis and predisposing hosts to various pathological conditions, including inflammatory bowel diseases, metabolic disorders, and cardiovascular diseases [8,9,10]. Such microbial imbalance may impair the production of critical metabolites, resulting in intestinal barrier dysfunction and exacerbating inflammatory responses that collectively threaten host health [11]. Therefore, the stability of intestinal microbial communities serves not only as a fundamental requirement for maintaining host homeostasis, but also as a crucial safeguard for overall health.

The intestinal microbial community plays an indispensable role in host health, with profound impacts on energy metabolism, immune function, and disease resistance [12,13]. Current evidence demonstrates that microbe-derived metabolites are mechanistically linked to the pathogenesis of diverse systemic conditions, including atherosclerosis, hypertension, heart failure, chronic kidney disease, hepatic disorders, diabetes mellitus, and neurological diseases [14]. Additionally, the intestinal microbiota demonstrated dynamic interactions with mammalian circadian metabolic rhythms [15]. The intimate connection between microbial ecology and host pathophysiology is exemplified by heart failure-induced intestinal barrier dysfunction [16]. Furthermore, the multifaceted interactions among intestinal microbes, their metabolic products, and the immune–endocrine–neural axes are implicated in conditions ranging from inflammatory bowel disease to cancer and major depressive disorder [17]. Importantly, the health-promoting effects of the intestinal microbiota are closely associated with their SCFA production, with dietary interventions emerging as a promising strategy to optimize microbial composition and restore immune homeostasis in disease states [18].

The liver serves as the central metabolic organ, orchestrating a myriad of enzymatic reactions that govern energy production, detoxification, drug metabolism, and physiological homeostasis [19]. The gut–liver axis represents a bidirectional communication network between the intestine and liver, wherein intestinal health modulates hepatic function, and liver-derived factors—including bile acids, cytokines, and immunomodulatory molecules—shape intestinal microbial composition and activity [20]. This axis operates via distinct anatomical and functional connections, primarily the biliary tract and portal vein, allowing the intestinal microbiota and its metabolites (e.g., SCFAs, secondary bile acids) to influence hepatic gene expression and immune regulation [21]. A homeostatic gut–liver axis supports liver health, whereas its disruption contributes to steatosis, fibrosis, and cirrhosis [22]. Consequently, investigation of the gut–liver axis in animal models provides critical insights into the functional mechanisms of intestinal microecosystems and their bidirectional interactions with host metabolic pathways.

Amphibians represent one of the most vulnerable vertebrate groups globally, with approximately 40% of species threatened by habitat destruction, climate change, and emerging infectious diseases [23,24]. Similarly, in amphibians adapting to terrestrial environments, the intestinal microbial community facilitates critical physiological functions such as digestion, immune modulation, osmoregulation, and metabolic balance maintenance [25]. For example, the intestinal microbiota of cave-dwelling *Oreolalax rhodostigmatus* tadpoles exhibited significantly higher proportions of *Lactobacillus*, *Cellulosilyticum*, and *Proteocatella*, which may enhance host nutrition and facilitate adaptation to the food-scarce cave environment [26]. Through the production of bioactive metabolites, the intestinal microbiome enhances amphibian adaptability to environmental challenges [27]. These functional contributions have established amphibian intestinal microbiota research as an international priority [28,29]. The Chong’an mustache toad (*Leptobrachium liui*), an endemic Chinese species within the family Megophryidae [30], has been primarily studied for its taxonomic classification [31], ethology [32], genomic characteristics [33,34], acoustic signaling [35,36] and thermal physiology [37]. However, critical knowledge gaps remain regarding its intestinal microbial composition, hepatic metabolomic profiles, and gut–liver axis interactions. The present study employed wild adult male *L. liui* specimens to investigate gut–liver axis interactions through integrated 16S rRNA gene sequencing and untargeted metabolomics. Our research objectives included (1) characterization of the microbial diversity in small and large intestinal segments, (2) identification of differentially abundant taxa and metabolites, and (3) correlation analysis between microbial communities and hepatic metabolic profiles. The overall aim was to reveal the interaction between functional changes in the intestinal microbiota and hepatic metabolism and to explore the influence of the intestinal microbiota on hepatic metabolic pathways and its role in maintaining organismal homeostasis.

## 2. Materials and Methods

### 2.1. Intestinal Content and Liver Tissue Sample Collection

**Figure 1:**
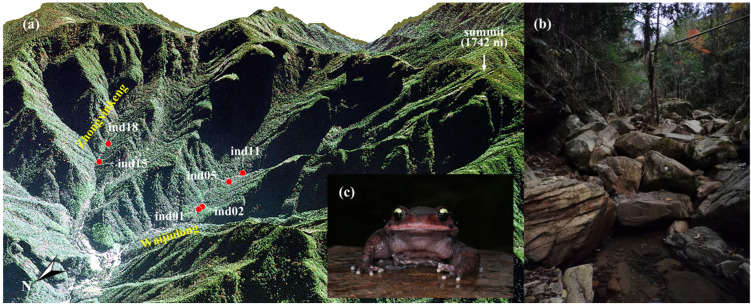
(**a**) Three-dimensional geographical distribution of the six sampling sites for adult male *Leptobrachium liui* in the Jiulongshan National Nature Reserve. The sampling sites are located at elevations between 720 and 939 m. Four specimens (ind01, ind02, ind05, ind11) were collected from streams in Waijiulong Valley, while two specimens (ind15, ind18) were collected from Zhongxinkeng Valley. (**b**) Habitat characteristics of the collection area. (**c**) Adult male *Leptobrachium liui*. Photographed by Guo-Hua Ding.

Six adult male *L. liui* were collected from two mountain valleys within the Jiulongshan National Nature Reserve (N 28.370°, E 118.887°, elevation 811.3 ± 82.0 m) in Suichang County, Zhejiang Province, eastern China, during mid-November 2021 (Figure 1). Four specimens (ind01, ind02, ind05, and ind11) were collected from Waijiulong Valley, while two specimens (ind15 and ind18) were collected from Zhongxinkeng Valley. Morphological data (snout-vent length: 87.7 ± 4.5 mm and body mass: 68.1 ± 8.2 g) and the environmental parameters of the sampling site were recorded, including dissolved oxygen (8.5 ± 0.9 mg/L), ambient humidity (81.8 ± 4.0%), water conductivity (16.7 ± 1.6 S/m), air temperature (13.2 ± 0.8 °C), and water temperature (13.9 ± 0.6 °C) (Table S1). No significant differences in these morphological and environmental parameters were observed between the two valleys (all *p* > 0.05; Table S1).

The adult individuals were transported to the Jiulongshan Field Research Center and euthanized by immersion in 1 L of tricaine methanesulfonate (MS-222) solution (400 ppm) for 15 min. Our protocol successfully achieved complete euthanasia in the *L. liui* specimens without requiring secondary methods. Death was confirmed by the absence of all reflexes, including righting response, breathing, and heartbeat. Dissections were performed under a stereomicroscope. The small intestine (SI) and large intestine (LI) were carefully removed, and their contents were extracted and transferred to sterile 1.5 mL RNase-free microcentrifuge tubes. Liver tissues were also collected in 1.5 mL RNase-free tubes. Subsequently, all samples were flash-frozen in liquid nitrogen for temporary storage before transport to the laboratory at Lishui University, China, where they were preserved at −80 °C in an ultra-low temperature freezer. DNA extraction, PCR amplification, and sequencing were completed within one month.

### 2.2. DNA Extraction and 16S rRNA Gene Sequencing

For microbial genomic DNA extraction, twelve intestinal content samples (six SI and six LI content samples from six individuals) were directly processed, and 150–200 mg aliquots were transferred to 2 mL microcentrifuge tubes. Total bacterial DNA was isolated from *L. liui* intestinal contents using the HiPure Stool DNA Kit (D3141, Magen Biotechnology, Guangzhou, China). DNA purity was assessed via NanoDrop 2000 spectrophotometry (Thermo Fisher Scientific, MA, USA) (A260/A280 ratio: 1.8–2.0), and integrity was verified by 1% agarose gel electrophoresis, confirming a distinct high-molecular-weight band (>15 kb) without smearing. The V3-V4 hypervariable regions of bacterial 16S rRNA genes were amplified from qualified DNA samples using the barcoded primers 341F (5′-CCTACGGGNGGCWGCAG-3′) and 806R (5′-GGACTACHVGGGTATCTAAT-3′). The PCR products were purified with an AxyPrep DNA Gel Extraction Kit (Corning Life Sciences, California, USA), quantified on an ABI StepOnePlus Real-Time PCR System, and subjected to high-throughput sequencing on an Illumina MiSeq PE250 platform (2 × 250 bp paired-end). The microbiota raw sequence data have been uploaded to NCBI’s Sequence Read Archive database (https://ncbi.nlm.nih.gov/sra (accessed on 30 June 2025); project no. PRJNA1265654).

### 2.3. Analysis of the Intestinal Microbial Data

To analyze the intestinal bacterial community of the male *L. liui*, the resulting raw sequencing data were first quality-filtered using FASTP (v0.20.0), with a quality threshold of Q20. Paired-end reads were then merged using FLASH (v1.2.11), with a minimum overlap of 10 bp. Chimeric sequences were identified and removed using the UCHIME algorithm to obtain high-quality clean data. High-quality sequences were clustered into operational taxonomic units (OTUs) at a 97% similarity threshold using the UPARSE algorithm implemented in USEARCH (v11.0). Taxonomic classification of representative OTU sequences was performed using the SILVA database (https://www.arb-silva.de (accessed on 9 June 2021), v138.1).

Taxonomic composition was analyzed at the phylum, order, and family levels, and taxa with relative abundances greater than 5% were considered dominant. Community composition analysis was performed, with the top 10 most abundant bacterial taxa (as determined by mean relative abundance) shown for each taxonomic rank (phylum, order, and family). Less abundant taxa (not in the top 10) were consolidated as ‘others’, and unassigned sequences were categorized as ‘unclassified’. We employed the non-parametric Kruskal–Wallis test for initial detection of features with different abundances, followed by the Wilcoxon rank-sum test for pairwise group comparisons. Linear discriminant analysis (LDA) was conducted as part of linear discriminant analysis effect size (LEfSe) analysis to identify significantly different functional taxa between the two groups. The LDA threshold was set to 4.0 to ensure the significance of the markers.

Alpha diversity indices (including the Sobs, Shannon, Simpson, and Pielou indices) were calculated using QIIME software (v2). Inter-group differences in alpha diversity were assessed using the Wilcoxon rank-sum test implemented in R (v4.0.3). For beta diversity analysis, we performed non-metric multidimensional scaling (NMDS) based on Bray–Curtis distances calculated from OTU abundance using the *vegan* (v2.6-4) package in R. This visualized structural differences in microbial communities between the two intestinal compartments. The statistical significance of between-group versus within-group dissimilarities was subsequently evaluated using analysis of similarities (ANOSIM).

### 2.4. Hepatic Metabolite Extraction and Quality Control (QC)

For metabolomic profiling, 100 mg of cryogenically homogenized (liquid nitrogen-pulverized) liver tissue from *L. liui* was aliquoted into a 1.5 mL microcentrifuge tube. Metabolites were extracted with 500 μL of 80% methanol/water by vortex mixing for 30 s, followed by ice-bath incubation (5 min) and centrifugation (20 min, 15,000× *g*, 4 °C). The supernatant was diluted with MS-grade water to a 53% methanol concentration, then recentrifuged under identical conditions. The supernatants were transferred to liquid chromatography–tandem mass spectrometry (LC-MS/MS) vials for analysis. Blank samples were prepared using 53% methanol/water as a surrogate matrix, undergoing identical processing procedures as for the experimental samples. Principal component analysis (PCA) was performed on QC samples using the prcomp function from the *stats* (v4.3.2) package in R to evaluate analytical variance and ensure system stability throughout the acquisition sequence. LC-MS/MS analysis was performed in both positive (POS) and negative (NEG) electrospray ionization modes to ensure comprehensive metabolite detection. The metabolome raw data have been deposited in OMIX, China National Center for Bioinformation/Beijing Institute of Genomics, Chinese Academy of Sciences (https://ngdc.cncb.ac.cn/omix (accessed on 30 June 2025); accession no. OMIX010255).

### 2.5. Identification and Analysis of Hepatic Metabolites

The raw metabolome data file was imported into Compound Discoverer 3.1 software for processing. Following initial screening, chromatographic peaks across different samples were aligned. Peak extraction was performed using predefined mass tolerance parameters, with subsequent quantification based on peak area integration. Targeted ions were then selected for further analysis. Molecular formula prediction was conducted utilizing both molecular ion peaks and fragment ions, with metabolite identification achieved through spectral matching against three major databases: MzCloud (https://www.mzcloud.org (accessed on 8 April 2021)), mzVault, and Masslist. To ensure data quality, background signals were systematically eliminated using blank samples, enabling reliable qualitative and quantitative metabolite analysis.

Metabolite classification and functional annotation were performed using the Kyoto Encyclopedia of Genes and Genomes (KEGG) database. Pathways related to ‘metabolism’ were screened, and their counts were statistically analyzed. A bar plot of metabolic pathways was constructed [38]. Based on the qualitative and quantitative analysis results of metabolites, the data from the POS and NEG modes were processed separately. First, unclassified metabolites were removed, and then the classified metabolite categories were statistically analyzed. The top 10 categories with the highest number of metabolites were retained, while the remaining categories were grouped as ‘other.’ Subsequently, data from both ionization modes were integrated, and a stacked bar plot of metabolite categories was generated. Furthermore, the mean expression levels of each metabolite across all samples were calculated by combining the POS and NEG mode data. Metabolites not identified in the MS2 database were removed, and the top 25 metabolites with the highest mean expression levels were selected and categorized. A bar plot of these top 25 metabolites was then generated [39].

### 2.6. Microbiota–Metabolome Correlation Analysis

Based on the SI and LI microbiota (at the family level) and hepatic metabolome analysis results, Pearson correlation coefficients between microbial taxa and metabolites were calculated using the *psych* (v 2.3.12) package in R. Statistical significance (*p* < 0.05) was determined through Fisher-Z transformation. Microbiota–metabolite pairs with absolute correlation coefficients > 0.05 were retained for further analysis. Following correlation analysis, redundant and unannotated metabolites were removed. Only metabolites classified under KEGG A class ‘metabolism’ and their corresponding correlated microbial taxa were selected. Using the abundance data of these microbiota in SI and LI contents along with the filtered metabolites, dynamic network heatmaps and correlation heatmaps were generated to visualize intestine-specific microbe–metabolite interactions.

### 2.7. Data Analyses

Statistical processing was implemented using R v4.3.2 (R Foundation for Statistical Computing, Vienna, Austria). All graphs and visualizations were generated using the *ggplot2* (v 3.5.0) and *ggcor* (v 0.9.7) packages in R. Descriptive statistics are reported as means ± standard deviations (SDs). Statistical inferences were accepted at a significance level of α = 0.05.

## 3. Results

### 3.1. Sequencing Data

The intestinal microbial communities of six male *L. liui* were characterized using high-throughput sequencing. The number of clean reads per sample ranged from 110,646 to 126,589 (mean ± SD: 119,362 ± 5821), generating a total of 1,389,151 high-quality effective tags across all samples, with sequence lengths spanning 234–440 base pairs. Cluster analysis revealed 4484 OTUs among the six samples, with individual sample OTU richness varying between 181 and 793 (Table S2). The dilution curves based on the Shannon index (Figure S1a) and the corresponding rank abundance curves (Figure S1b) confirmed adequate sequencing depth and indicated similar levels of microbial richness among the groups. The Venn diagram showed that the SI and LI of *L. liui* contained 1029 and 929 OTUs, respectively, with 227 OTUs shared between both intestinal segments (Figure S2), indicating distinct microbial community compositions between the two intestinal compartments of *L. liui*.

### 3.2. Microbial Composition Between the Two Intestinal Compartments

**Figure 2:**
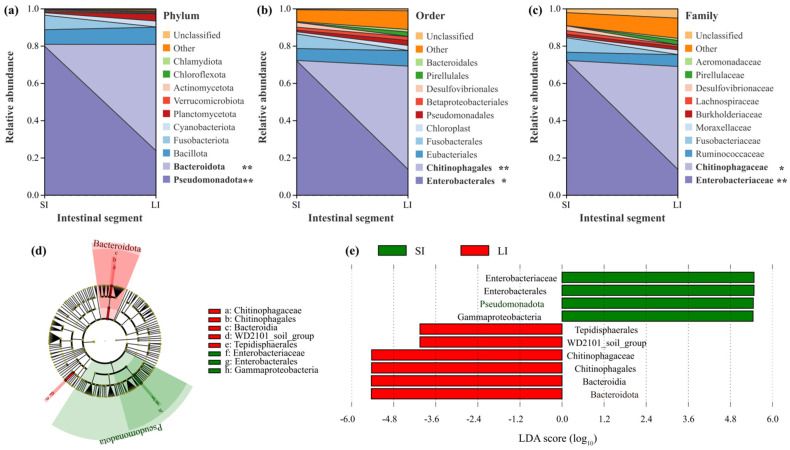
Bacterial community composition and differentially abundant taxa between the small intestine (SI) and large intestine (LI) of adult male *Leptobrachium liui*. Relative abundance of bacterial taxa at the (**a**) phylum, (**b**) order, and (**c**) family levels. Asterisks indicate significantly different taxa between intestinal segments (*: *p* < 0.05, **: *p* < 0.01). (**d**) Cladogram generated from the LEfSe analysis illustrating the phylogenetic distribution of bacterial lineages with significant differences between the SI and LI. (**e**) Linear discriminant analysis (LDA) scores of significantly enriched bacterial taxa in the SI (green bars) and LI (red bars). Only taxa meeting the threshold criterion of LDA score > 4.0 are shown.

The V3-V4 region of bacterial 16S rRNA genes from the intestinal microbiota of *L. liui* was sequenced for microbial characterization. Taxonomic composition was analyzed based on OTU annotation. At the phylum level, six phyla exceeded 5% relative abundance in both intestinal segments. In the SI, Pseudomonadota (79.95%), Bacillota (7.83%), and Fusobacteriota (7.78%) dominated, collectively representing > 95% of the microbiota. In the LI, Bacteroidota (56.99%), Pseudomonadota (23.91%), and Bacillota (9.26%) were predominant, accounting for > 90% abundance. Wilcoxon rank-sum test revealed significant differences (*p* < 0.05) in the proportions of Pseudomonadota (decreasing from SI to LI) and Bacteroidota (increasing from SI to LI) (Figure 2a).

At the order level, six orders showed > 5% abundance. The SI was dominated by Enterobacterales (72.14%), Fusobacterales (7.78%), and Eubacteriales (6.43%) (collectively > 86%), while Chitinophagales (55.36%), Enterobacterales (13.93%), and Eubacteriales (8.30%) prevailed in the LI (> 77% combined). Significant differences (*p* < 0.05) were observed for Chitinophagales (markedly higher in LI), with Enterobacterales showing marginal significance (*p* < 0.1) (Figure 2b).

At the family level, four families exceeded 5% abundance. Enterobacteriaceae (72.14%) and Fusobacteriaceae (7.77%) dominated SI contents (> 79% combined), whereas Chitinophagaceae (55.16%), Enterobacteriaceae (13.93%), and Ruminococcaceae (6.30%) were predominant in LI contents (> 75% total). Significant differences were found for Enterobacteriaceae (*p* < 0.05, higher in SI) and Chitinophagaceae (*p* < 0.05, substantially higher in LI) (Figure 2c).

LEfSe analysis (LDA score > 4.0) revealed distinct microbial enrichment patterns between intestinal segments. The LI microbiota was significantly enriched in Chitinophagaceae, Bacteroidia, and Bacteroidota, while SI was preferentially enriched in Enterobacterales and Gammaproteobacteria, suggesting compartment-specific functional specialization of the intestinal microbiota (Figure 2d,e).

### 3.3. Microbial Diversity Between the Two Intestinal Compartments

**Figure 3:**
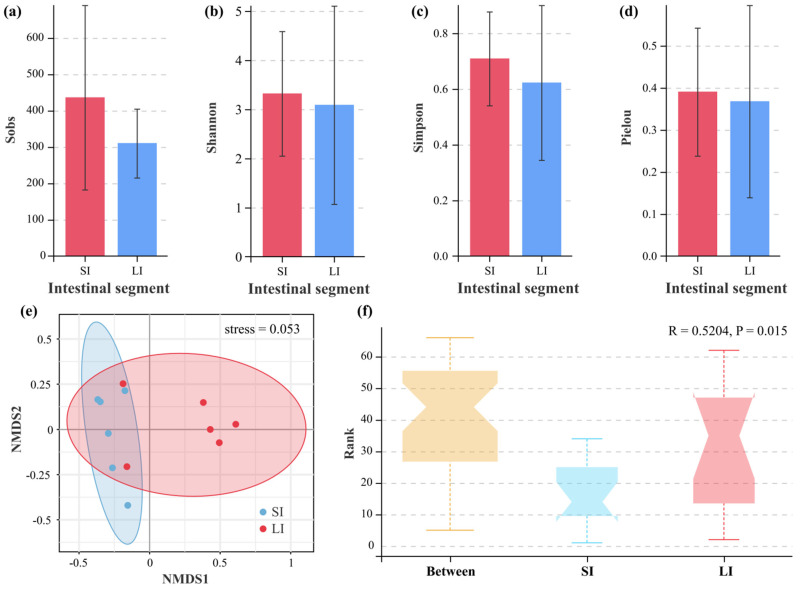
Alpha and beta diversity of bacterial communities in the small intestine (SI) and large intestine (LI) of adult male *Leptobrachium liui*. Alpha diversity indices: (**a**) Sobs index, (**b**) Shannon index, (**c**) Simpson index, and (**d**) Pielou index. (**e**) Non−metric multidimensional scaling (NMDS) plot based on Bray−Curtis dissimilarity showing differences in bacterial community structure between the SI and LI, with a stress value of 0.053. (**f**) Analysis of similarities (ANOSIM) demonstrating significant differences in bacterial community composition between and within the SI and LI groups (R = 0.5204, *p* = 0.015).

At the OTU level, four standard alpha diversity indices (Sobs, Shannon, Simpson, and Pielou) were calculated to assess species richness and evenness. No statistically significant differences were observed between the SI and LI for any of these metrics (*p* > 0.05, Mann–Whitney U test), suggesting comparable microbial diversity in both intestinal compartments despite their distinct taxonomic compositions (Figure 3a–d). Beta diversity analyses (NMDS) revealed distinct clustering patterns between SI and LI microbial communities (stress = 0.053, Figure 3e). Furthermore, the ANOSIM test revealed significantly greater dissimilarities between intestinal segments than within segments (R = 0.5204, *p* = 0.015), demonstrating significant differences in microbial community structures between the two intestinal compartments (Figure 3f).

### 3.4. QC Assessment for Metabolomic Analysis

PCA of the QC samples demonstrated system stability throughout the metabolomic analysis in both ionization modes (Figure S3). QC samples clustered tightly in both the POS (Figure S3a, PC1: 29.2%, PC2: 20.7%) and NEG (Figure S3b, PC1: 27.1%, PC2: 21.6%) modes, confirming good analytical reproducibility and minimal technical variation. The control samples displayed expected biological variation, with greater dispersion across the PCA plots while maintaining clear separation from the QC samples. This indicates that the analytical platform reliably captured biological differences without significant interference from technical factors.

### 3.5. Hepatic Metabolome Characterization and Quantification

A total of 1438 metabolites were detected in POS mode (1251 with MS2 and 187 with MS1 data), while 796 metabolites were detected in NEG mode (704 with MS2 and 92 with MS1 data), all of which were successfully annotated.

**Figure 4:**
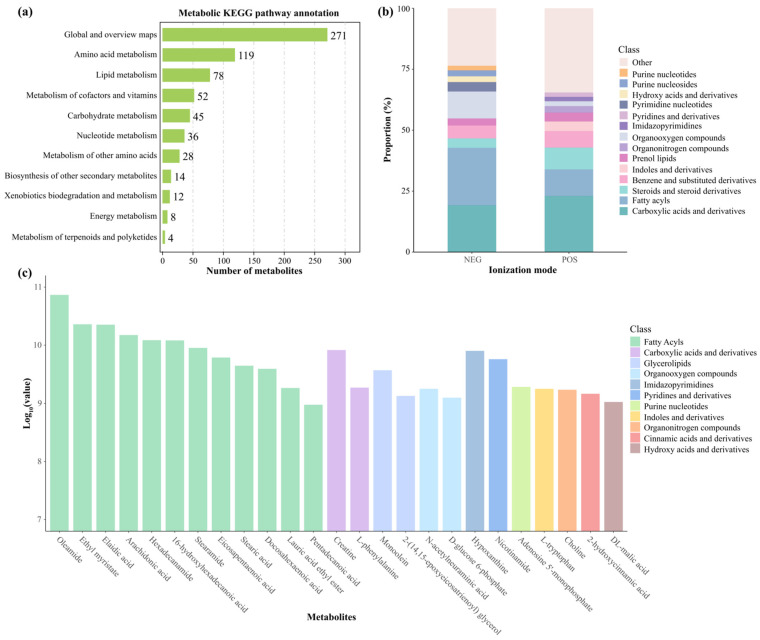
Metabolic profiling of hepatic metabolites in adult male *Leptobrachium liui*. (**a**) Distribution of identified metabolites across KEGG metabolic pathways. Numbers indicate the counts of metabolites in each pathway. (**b**) Relative proportions of metabolite classes detected in negative (NEG) and positive (POS) ionization modes using LC−MS/MS. (**c**) Top 25 most abundant metabolites based on mean intensity values (log_10_ scale), colored by their respective chemical classes.

Our metabolic classification and statistical analysis revealed 667 metabolism-associated pathways in hepatic samples from *L. liui*. Global and overview maps constituted the most abundant category (271 pathways), followed by amino acid metabolism (119 pathways) and lipid metabolism (78 pathways) (Figure 4a). Metabolomic analysis of the *L. liui* samples revealed distinct metabolite class distributions between the POS and NEG modes (Figure 4b). In POS mode, carboxylic acids and derivatives represented the most abundant metabolite class, followed by fatty acyls, steroids, and steroid derivatives. NEG-mode profiling showed fatty acyls as the predominant class, with carboxylic acids and derivatives and organooxygen compounds also exhibiting significant representation. Analysis of the top 25 most abundant metabolites in both ionization modes identified fatty acyls, carboxylic acids and derivatives, and glycerolipids as the major classes, with oleamide showing the highest mean expression level, and DL-malic acid the lowest (Figure 4c).

### 3.6. Integrated Analysis of Intestinal Microbiota and Hepatic Metabolome

**Figure 5:**
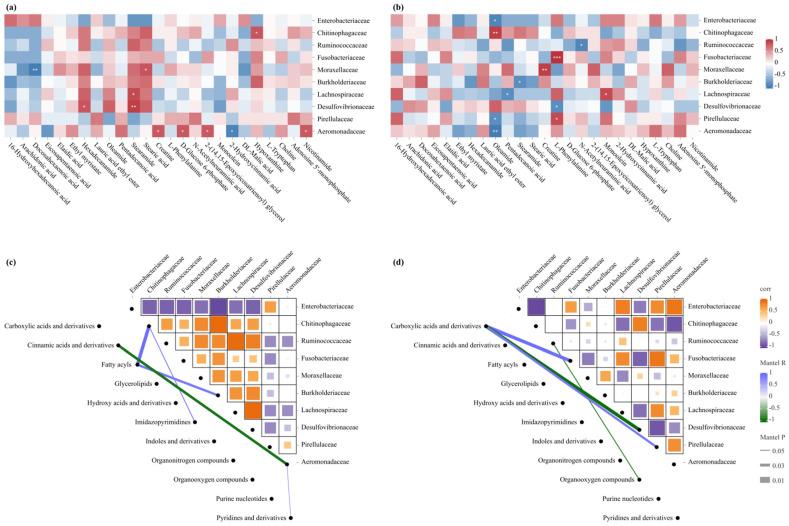
Correlation analysis between bacterial families and metabolites in adult male *Leptobrachium liui*. Heatmaps of Spearman correlation between the relative abundances of predominant bacterial families and top metabolites in the (**a**) small and (**b**) large intestines. *: *p* < 0.05, **: *p* < 0.01, ***: *p* < 0.001. Network-based visualization of significant correlations between bacterial families and metabolite classes in the (**c**) small and (**d**) large intestines. The line thickness in the network plots represents different significance thresholds, as indicated in the legend.

Integrated analysis of the intestinal microbiota and hepatic metabolome revealed significant microbe–metabolite correlations with distinct intestinal segment-specific patterns (Figure 5). In fatty acid metabolism, the SI displayed complex patterns: Aeromonadaceae positively correlated with 2-(14,15-epoxyeicosatrienoyl) glycerol, and Desulfovibrionaceae positively associated with fatty acid amides (hexadecanamide and stearamide), whereas Moraxellaceae exhibited contrasting fatty acid associations (positive with stearic acid, negative with docosahexaenoic acid) (Figure 5a). The LI exhibited different patterns, with oleamide showing divergent correlations across families (negative: Enterobacteriaceae, Pirellulaceae, Aeromonadaceae; positive: Chitinophagaceae) and stearamide negatively associating with Burkholderiaceae (Figure 5b). In energy and amino acid metabolism, the SI showed Aeromonadaceae positively correlating with energy-related metabolites (creatine, D-glucose 6-phosphate) and nicotinamide, but negatively with 2-hydroxycinnamic acid (Figure 5a). The LI displayed different patterns: L-phenylalanine showed positive associations with Fusobacteriaceae and Pirellulaceae but negative correlation with Desulfovibrionaceae, and creatine positively correlated with Moraxellaceae (Figure 5b). Other metabolite correlations included hypoxanthine with Chitinophagaceae in the SI and monoolein with Lachnospiraceae and N-acetylneuraminic acid with Ruminococcaceae in the LI (all *p* < 0.05) (Figure 5a,b).

Network analysis further revealed distinct correlations between bacterial families and metabolites in different intestinal segments. In the SI, both Chitinophagaceae and Burkholderiaceae showed positive correlations with fatty acids, with the former additionally correlating with imidazopyrimidines; Aeromonadaceae negatively correlated with cinnamic acids and derivatives but positively associated with pyridines and derivatives (Figure 5c). In the LI, Ruminococcaceae displayed negative correlation with organooxygen compounds, while Pirellulaceae and Fusobacteriaceae showed positive associations with carboxylic acids and derivatives; conversely, Desulfovibrionaceae exhibited significant negative correlation with this metabolite class (Figure 5d).

## 4. Discussion

This study revealed that, while alpha diversity remained consistent between intestinal segments of *L. liui*, the SI and LI harbored significantly divergent microbial community structures with distinct metabolite interaction patterns. Despite specimens being collected from two different mountain valleys within the nature reserve, no significant environmental or morphological differences were detected between sampling sites, indicating that the observed microbial and metabolic differences reflect intrinsic biological variation rather than location-based factors. The oxygen-rich SI environment favors aerotolerant microorganisms such as Pseudomonadota and Bacillota, which contribute to energy metabolism, intestinal barrier maintenance, immune regulation, pathogen resistance, antioxidant activity, and modulation of host metabolic and neurological functions [40]. Bacillota, meanwhile, appear to modulate host immune tolerance and resistance mechanisms to provide pathogen defense without excessive immunopathology [41]. In contrast, the anaerobic LI promotes Bacteroidota dominance, supporting short-chain fatty acid production, intestinal barrier integrity, anti-inflammatory activity, and metabolic regulation [42]. However, our study represents a snapshot from a limited sample size (*n* = 6) and geographic range, which may not fully capture the natural variation in this specie’s intestinal microbiota.

These findings support segment-specific organization of intestinal microbiota in *L. liui*. The distinct microbial communities and metabolite interaction patterns between the SI and LI may reflect functional specialization adapted to different physiological environments [43]. However, the adaptive significance of this segmental organization and its role in amphibian physiology remain to be determined through experimental studies.

Our analysis identified several critical microbial–metabolite interactions that may be relevant to amphibian physiology, though their specific functional significance requires experimental validation. Importantly, these correlations do not establish causality, and the underlying mechanisms remain to be determined through controlled experiments. Mountain-dwelling amphibians like *L. liui* may face physiological challenges including seasonal temperature fluctuations, variable water chemistry, and food scarcity. Such conditions could select for specialized metabolic adaptations, particularly in fatty acid, energy and amino acid metabolism [44], though the specific adaptations in *L. liui* require further investigation.

We observed divergent correlation patterns between the intestinal microbiota and hepatic metabolites across segments, with predominantly positive correlations in the SI and more complex patterns in the LI. This segment-specific distribution aligns with evidence that site-specific intestinal microbiota–host interactions are critical for functional outcomes [45], as exemplified by the differential correlation patterns of Aeromonadaceae between intestinal segments.

Key bacterial families and metabolites identified in our analysis have established physiological roles in other biological systems. For example, oleamide functions in regulation and neural signaling [46], while bacterial families such as Enterobacteriaceae [47], Aeromonadaceae [48], and Fusobacteriaceae [49] are known to influence host metabolism in various organisms. Similarly, L-phenylalanine [50] serves as an essential amino acid in protein synthesis. The observed correlations suggest possible metabolic associations that may contribute to amphibian physiology, though the specific regulatory mechanisms and adaptive significance require experimental validation.

Network analysis revealed complex correlation patterns between intestinal microbiota and metabolites, with both positive and negative associations suggesting diverse regulatory mechanisms that collectively influence host metabolic physiology. Our findings revealed significant associations between intestinal microbial communities and specific metabolite classes in both intestinal segments. In the SI, Chitinophagaceae showed positive correlations with fatty acyls and imidazopyrimidines, potentially reflecting involvement in fatty acid-related metabolic processes. While certain Chitinophagaceae members can degrade complex carbohydrates and influence host metabolic pathways [51], their specific role in fatty acid metabolism remains unclear. Similarly, Burkholderiaceae displayed positive correlations with fatty acyls, though current evidence is insufficient to establish a direct link between this family and lipid metabolism [52]. Aeromonadaceae exhibited a negative correlation with cinnamic acids and derivatives but a positive correlation with pyridines and derivatives, suggesting complex metabolic interactions [53]. Notably, both cinnamic acids [54] and pyridine derivatives [55] possess demonstrated antimicrobial, antiviral, anti-inflammatory, and neuropharmacological activities.

In the LI, Ruminococcaceae showed a negative correlation with organooxygen compounds. Given that Ruminococcaceae is associated with improved lipid metabolism [56], and organooxygen compounds participate in key metabolic pathways, including glycolysis and energy metabolism [57]; this negative correlation may suggest competitive interactions in metabolic regulation. Both Pirellulaceae and Fusobacteriaceae demonstrated positive correlations with carboxylic acids and derivatives, possibly reflecting complex microbial interactions or host metabolic environment changes, though direct mechanistic evidence remains lacking. Desulfovibrionaceae, which is known to produce H_2_S through sulfate reduction and is associated with inflammatory bowel disease [58], showed a negative correlation with carboxylic acids and derivatives. This pattern may reflects its involvement in sulfate reduction pathways that could influence intestinal redox status.

In conclusion, our findings reveal segment-specific organization of microbial communities in the amphibian intestine with distinctive metabolite interaction patterns. While these correlations suggest potential functional relationships, experimental validation is needed to establish causality and biological significance. The identified patterns provide both fundamental insights into amphibian gut–liver axis function and potential tools for conservation monitoring, establishing a foundation for understanding intestinal microbiota contributions to amphibian physiology.

## 5. Conclusions

This study reveals that *L. liui* exhibits sophisticated segment-specific intestinal microbiota organization with distinct metabolite interaction networks. The contrasting microbial structures between SI and LI, combined with segment-specific correlation patterns, indicate functional specialization that may reflect adaptation to different physiological environments. These intestinal microbiota–hepatic metabolite associations suggest potential responses to environmental challenges faced by mountain-dwelling amphibians. While causality requires experimental validation, our findings provide insights into amphibian gut–liver axis function and potential biomarkers for population monitoring. This work establishes a foundation for understanding intestinal microbiota contributions to amphibian physiology.

## Data Availability

All 16S rRNA gene sequences obtained in this study have been de posited in the NCBI Sequence Read Archive (https://www.ncbi.nlm.nih.gov/bioproject (accessed on 30 June 2025); project no. PRJNA1265654). The metabolome raw data have been deposited in OMIX, China National Center for Bioinformation/Beijing Institute of Genomics, Chinese Academy of Sciences (https://ngdc.cncb.ac.cn/omix (accessed on 30 June 2025); accession no. OMIX009902). The 16S rRNA and liver metabolite matrix datasets are available on Figshare https://doi.org/10.6084/m9.figshare.28418264.v1.

## Supplementary Materials

The following supporting information can be downloaded at: https://www.mdpi.com/article/10.3390/ani15131973/s1, Figure S1: (a) Dilution curve of the Shannon index and (b) the corresponding rank abundance curve for all samples collected from the contents of the small intestine (SI) and large intestine (LI) in adult male *Leptobrachium liui*, determined by high-throughput sequencing; Figure S2: Venn diagram of the numbers of identified microbial OTUs between the small intestine (SI) and large intestine (LI) in adult male *Leptobrachium liui*; Figure S3: Principal component analysis of metabolomic profiles from the sample and quality control (QC) groups in (a) positive (POS) and (b) negative (NEG) ionization modes; Table S1: Morphological data and environmental parameters of six adult male *Leptobrachium liui* specimens collected from Jiulongshan National Nature Reserve, Zhejiang Province, China in November 2020. SVL: snout-vent length, BM: body mass, LWM: liver wet mass, DO: dissolved oxygen, COND: water conductivity, AT: air temperature, WT: water temperature, HUM: ambient humidity. *p*-values were calculated using the Wilcoxon rank-sum test comparing specimens from Waijiulong Valley (*n* = 4) and Zhongxinkeng Valley (*n* = 2). Statistical significance was set at α = 0.05; Table S2: Overview of the sequencing data for operational taxonomic units (OTUs) from the small intestine (SI) and large intestine (LI) of adult male *Leptobrachium liui*.
