# Supplementary material for: Integrated Microbiome–Metabolome Analysis Reveals Intestine–Liver Metabolic Associations in the Moustache Toad
Source: Animals (Basel). 2025 Jul 4;15(13):1973. doi: 10.3390/ani15131973 (PMC12248546; doi:10.3390/ani15131973)
Supplement: Supplementary file 1 [file animals-15-01973-s001.zip › animals-3683747-supplementary.pdf]

Supplemental files

Integrated Microbiome – Metabolome Analysis Reveals Intestine – Liver Metabolic Associations in the Moustache Toad

Shui-Sheng Yu, Jing-Wen Xiang, Lin Zhang, Xiao-Hua Guo, Yu Wang, Guo-Hua Ding, Hua-Li Hu

Table S1 Morphological data and environmental parameters of six adult male *Leptobrachium liui* specimens collected from Jiulongshan National Nature Reserve, Zhejiang Province, China in November 2020. SVL: snout-vent length, BM: body mass, LWM: liver wet mass, DO: dissolved oxygen, COND: water conductivity, AT: air temperature, WT: water temperature, HUM: ambient humidity. P-values were calculated using Wilcoxon rank-sum test comparing specimens from Waijiulong valley (n=4) and Zhongxinkeng valley (n=2). Statistical significance was set at $\alpha = 0.05$.

Mountain valley	Individual ID	Morphological data			Environmental parameters of the sampling site				
		SVL (mm)	BM (g)	LWM (g)	DO (mg/L)	COND (μS/cm)	AT (°C)	WT (°C)	HUM (%)
Waijiulong (n = 4)	ind01	84.12	62.43	1.54	8.04	18	13.6	14.5	77.4
	ind02	95.61	74.00	1.41	9.56	18	14.1	14.7	79.6
	ind05	86.17	58.56	0.93	8.52	14	13.8	14.2	80.00
	ind11	81.84	60.58	1.26	9.66	18	11.6	13.5	89.8
Zhongxinkeng (n = 2)	ind15	87.5	71.36	1.06	8.01	15	13.2	13.5	82.5
	ind18	90.84	81.44	1.10	7.17	17	13.1	13	81.2
P-value in Wilcoxon rank-sum test		0.486	0.247	0.486	0.105	0.461	0.488	0.159	0.488

Table S2 The overview of sequencing data of operational taxonomic units (OTUs) from small intestine (SI) and large intestine (LI) in adult male *Leptobrachium liui*.

Intestinal segment	Sample ID	Raw reads	Effective tags	Effective ratio (%)	Min length (bp)	Max length (bp)	OTUs
SI	SI01	135,521	121,622	89.74	403	432	409
	SI02	130,412	118,238	90.66	403	431	237
	SI05	126,258	113,804	90.14	241	430	399
	SI11	126,955	116,570	91.82	244	430	181
	SI15	121,266	108,922	89.82	403	431	701
	SI18	128,570	115,810	90.08	260	440	793
LI	LI01	127,487	113,071	88.69	282	431	241
	LI02	120,481	108,600	90.14	325	430	187
	LI05	135,613	122,607	90.41	267	431	299
	LI11	122,870	109,635	89.23	234	430	406
	LI15	132,220	117,781	89.08	304	430	253
	LI18	135,439	122,491	90.44	241	433	378

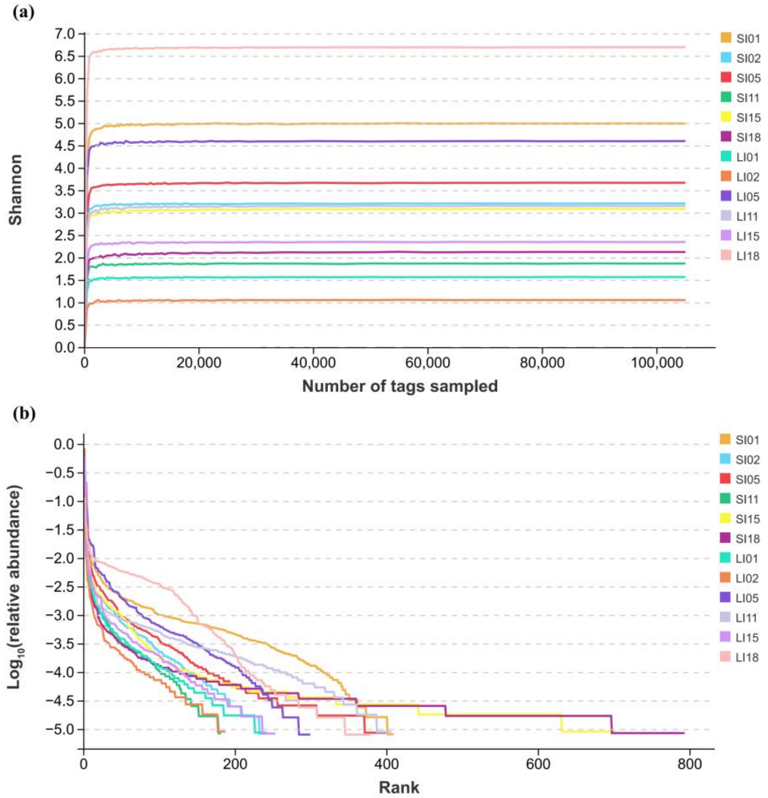

Figure S1 (a) The dilution curve of the Shannon index and (b) the corresponding rank abundance curve for all samples collected from the contents of small intestine (SI) and large intestine (LI) in adult male *Leptobrachium liui,* determined by high-throughput sequencing.

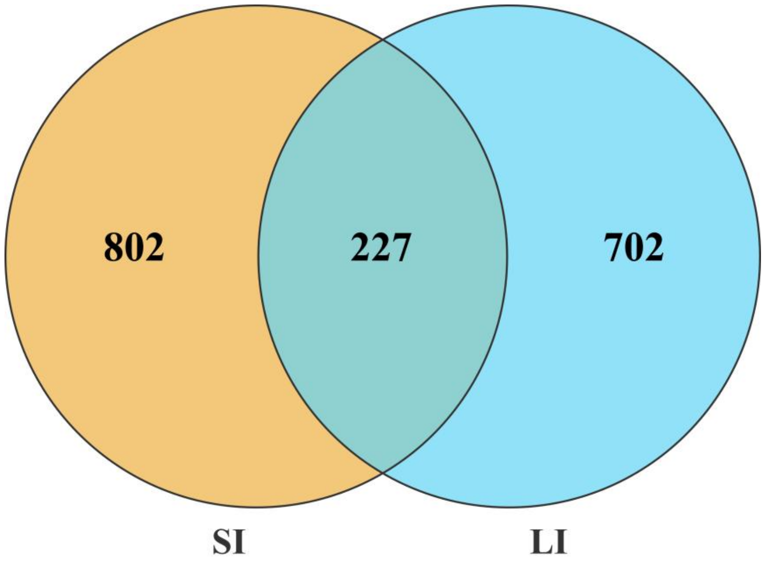

Figure S2 The Venn diagram for the numbers of identified microbial OTUs between small intestine (SI) and large intestine (LI) in adult male *Leptobrachium liui*.

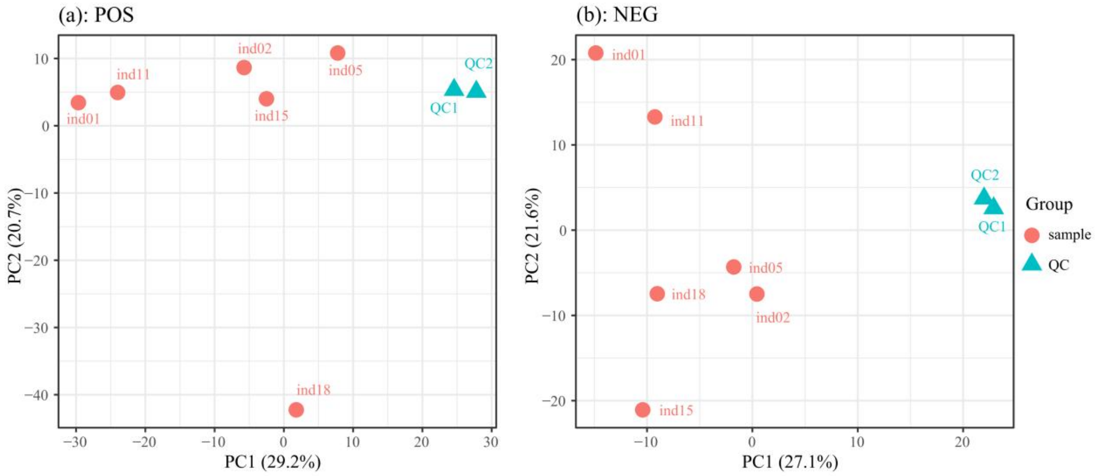

Figure S3 Principal component analysis of metabolomic profiles from samples and quality control (QC) groups in (a) positive (POS) and (b) negative (NEG) ionization modes.
